# Transcriptomic analysis of tumor tissues and organoids reveals the crucial genes regulating the proliferation of lung adenocarcinoma

**DOI:** 10.1186/s12967-021-03043-6

**Published:** 2021-08-26

**Authors:** Xiao Ma, Su Yang, Hesheng Jiang, Yujie Wang, Zhen Xiang

**Affiliations:** 1 Fudan University Shanghai Cancer Center, 270 Dong-An Road, Shanghai, 200032 China; 2 Department of Thoracic Surgery, Ruijin Hospital, Shanghai Jiaotong University School of Medicine, Shanghai, 200025 China; 3 Department of Surgery, Oregon Health & Science University, Portland, OR USA; 4 Department of Radiation Oncology, Ruijin Hospital, Shanghai Jiaotong University School of Medicine, Shanghai, 200025 China

**Keywords:** CCNB2, CDC25A, CDK1, Lung adenocarcinoma, Organoid

## Abstract

**Background:**

Accumulative evidence shows that an organoid is a more practical and reliable tool in cancer biology research. This study aimed to identify and validate crucial genes involved in non-small cell lung cancer carcinogenesis and development using the transcriptomic analysis of tumor tissues and organoids.

**Methods:**

Gene set enrichment analysis (GSEA) of tumor tissues, tumor organoids, and normal tissues was performed to reveal the similar and different mechanisms involved in lung adenocarcinoma (LUAD) and lung squamous cell carcinoma (LUSC) carcinogenesis and progression. Differentially expressed gene analysis, prognostic analysis, and gene co-expression network analysis were further used to identify hub genes involved in LUAD and LUSC carcinogenesis and development. Finally, LUAD cell lines and organoids were used to validate these findings.

**Results:**

GSEA analysis was performed to reveal the similar mechanisms involved in LUAD and LUSC carcinogenesis and development, such as P53 signaling pathway, base mismatch repair, DNA replication, cAMP signaling pathway and PPAR pathway. However, comparing with LUSC organoids, LUAD organoids showed downregulation of immune-related pathways, inflammation-related pathways, MAPK signaling pathways, and Rap1 signaling pathways, although these pathways were downregulated in LUAD and LUSC tissues by comparing with normal lung tissues. Further gene co-expression network analysis and prognostic analysis indicated CDK1, CCNB2, and CDC25A as the hub tumor-promoting genes in LUAD but not in LUSC, which were further validated in other datasets. Using LUAD cell lines and organoid models, CDK1 and CCNB2 knockdown were found to suppress LUAD proliferation. However, CDC25A knockdown did not inhibit LUAD cell line proliferation but could effectively suppress LUAD organoid growth, indicating that an organoid could be used as an effective tool to study cancer biology in LUAD.

**Conclusions:**

The results revealed CDK1, CCNB2, and CDC25A as the hub genes involved in LUAD carcinogenesis and development, which could be used as the potential biomarkers and targets for LUAD.

**Supplementary Information:**

The online version contains supplementary material available at 10.1186/s12967-021-03043-6.

## Introduction

Lung cancer is the leading cause of cancer-related death worldwide, with an estimated 1.8 million deaths each year [1]. Non-small cell lung cancer (NSCLC), the most common subtype with 85% of all cases, has an overall 5-year survival rate of 17.8%; more than half of patients die within 1 year [2]. NSCLC can be categorized into squamous cell carcinoma, adenocarcinoma, large-cell lung cancer, and small cell lung cancer [3, 4]. Recently, the treatment targeting somatic mutations and PD-L1/PD-1 improved survival, but most patients with NSCLC did not respond to or developed resistance to these treatments [5]. Therefore, new potential biomarkers need to be urgently discovered to improve NSCLC diagnosis, treatment, and prognosis.

Organoids are self-organizing 3D structures grown from stem cells, which can recapitulate the essential aspects of organ structure and function [6]. Organoids established from lung cancer tissues retain tumor histopathology as well as gene mutations, copy number aberrations, and gene expression profiles; hence, they are used for therapeutic screening, including chemotherapy, FGFR and MEK-targeted therapies, and immune checkpoint inhibitors [6–9]. Tumor organoid is a relatively ideal model to bridge the gaps between cell lines and animal and clinical research. Chen et al. indicated that using tumor organoids for studying cancer biology could help reveal more invasion-driver genes previously undescribed [10]. Corte et al. found that the dual blockade of MEK and PD-L1 in ex vivo models based on organoid culture showed synergistic anti-tumor activity by enhancing anti-tumor immune reaction and recruiting immune cells to the tumor sites [8].

Pure lung cancer cell clusters not containing stromal components were obtained by culturing organoids. Therefore, the mechanisms involved in carcinogenesis and tumor progression of lung cancer could be well studied using lung cancer organoid models [11]. Previous studies extensively used cancer-associated databases, such as The Cancer Genome Atlas, Gene Expression Omnibus, and Kaplan–Meier Plotter database [12–15]. However, these studies were performed using the transcriptomic data of tumor tissues. This study aimed at performing the integrated transcriptomic analysis of tissues and organoids in LUAD and LUSC, respectively. Then, cyclin-dependent kinase 1 (CDK1), cyclin B2 (CCNB2), and cell division cycle 25A (CDC25A) were further screened and identified as the hub genes involved in LUAD carcinogenesis and development, but not in LUSC. Finally, in vitro experiments were designed using cancer cell lines and cancer organoids to validate these findings. The findings of this study might help identify the potential biomarkers and therapeutic targets for LUAD.

## Materials and methods

### Data acquisition from The Cancer Genome Atlas and Gene Expression Omnibus databases

The transcriptomic data used in this study was assessed and downloaded from The Cancer Genome Atlas (TCGA) database (https://portal.gdc.cancer.gov/) and the Gene Expression Omnibus (GEO) database (GSE119004, https://www.ncbi.nlm.nih.gov/geo/). The TCGA database contained the data of 525 LUADs with 58 paired normal tissues and 501 LUSCs with 49 paired normal tissues. The GEO project GSE119004 contained the transcriptomic data of two paired LUSC tissues and organoids and six paired LUAD tissues and organoids.

### Gene set enrichment analysis

GSEA software and MSigDB gene sets were downloaded from the GSEA database (https://www.gsea-msigdb.org/gsea/index.jsp). GSEA analysis was performed following the instructions, and the results were displayed by plotting a bubble chart using the “ggplot2” package (Version 3.2.1) in R software (Version 3.5.0).

### Kaplan–Meier plotter database analysis on survival in lung cancer

Survival analysis data of patients with lung cancer were searched in the Kaplan–Meier Plotter database (http://kmplot.com/analsysis/) according to the following criteria: (1) cancer: lung cancer; (2) gene: RAD51, CDK1, CCNB2, CDC25A, and GTSE1; and (3) survival: overall survival (OS).

### Gene co-expression network analysis

First, the “corrplot” package (Version 0.84) in R software (Version 3.5.0) was used to calculate the Spearman correlation coefficient between two genes. Then, the gene pairs with coefficient > 0.3 and *P* < 0.001 were used to establish the gene co-expression network analysis with Cytoscape software. Finally, the genes with degrees ≥ 18 were identified as the hub genes involved in LUAD or LUSC carcinogenesis and progression.

### Western blot analysis

Harvested cultured organoids and cells were lysed in lysis buffer (P0013K, Beyotime, Shanghai, China) containing 1% PMSF. The concentrations of proteins of lysates were quantified using a BCA protein assay kit (P0012S, Beyotime), and these proteins were loaded in each lane. The protein extracts were separated by 10% SDS-PAGE (30 μg/lane) and transferred to a polyvinylidene fluoride membrane. Rabbit polyclonal CDK1 (A12414, Abclonal, Wuhan, China), CCNB2 (A3352, Abclonal), and CDC25A (A1173, Abclonal) were used at a dilution of 1:1000, and the HRP-conjugated GAPDH monoclonal antibody (HRP-60004, Proteintech, Wuhan, China) was used at 1:5000 dilution. Finally, the protein expression was detected using an electrochemiluminescence (ECL) Western blot detection system.

### Cell line and cell culture

The LUAD cell lines A549, PC9, and H2030 were purchased from ATCC (MD, USA). These LUAD cell lines were maintained in the RPMI 1640 medium supplemented with 10% fetal bovine serum (Thermo Fisher Scientific, MA, USA) at 37 °C in a humid incubator with 5% CO_2_.

### Tumor tissue processing and organoid establishment

Three or four pieces of 1-mm^3^ LUAD tissues were separated within 1 h of removal from the patients enrolled in Department of Thoracic Surgery, Ruijin Hospital, Shanghai Jiaotong University School of Medicine, and placed in cold Hank’s balanced salt solution with antibiotics (penicillin: 200 U/mL; streptomycin: 0.2 mg/mL; primocin: 200 μg/mL). The written informed consents were signed by the three patients. A paired lung normal (Normal-1) and LUAD tissue (LUAD-1), another LUAD tissue (LUAD-2) and a malignant pleural effusion sample of LUAD patient (LUAD-3) were collected from three LUAD patients. These tissues were cut after washing using PBS with antibiotics (penicillin: 200 U/mL; streptomycin: 0.2 mg/mL; primocin: 200 μg/mL). Then, these tissues were transferred to the EP tubes, and 1 mL of DMEM with collagenase IV (1.5 mg/mL) and hyaluronidase (20 μg/mL) was added. The tissues were incubated in a water bath at 37 °C, and the EP tubes were vibrated using a vortex. The suspensions were passed through 100-μm cell strainers (BD Falcon, CA, USA). The strained cells were centrifuged at 1000 rpm for 5 min and resuspended in 80 μL of DMEM. Further, 80 µL of matrix Matrigel was added and mixed well. The mixture was seeded in a 24-well plate (60–80 µL /well). After incubation in the incubator at 37 °C for 20 min, the LUAD organoid culture medium purchased from OmaStem (OmaStem® Lung Cancer Medium (Human), OM14, Guangzhou, China) was added (800–1000 µL /well). The medium was changed every 5 days. For passage, the medium was removed, 1 mL of TrypLE Express Enzyme (12604021, Thermo Scientific, MA, USA) was added to digest LUAD organoids, and the cells were resuspended for organoid passages.

### Hematoxylin–eosin staining and immunohistochemistry

The LUAD tissues and harvested LUAD organoids were fixed with 10% formalin and embedded in paraffin. Four-micrometer-thick sections were made for hematoxylin–eosin (H&E) staining and immunohistochemistry (IHC). For IHC, the sections were first deparaffinized and hydrated. The slides were then immersed in boiling 10 mmol/L sodium citrate buffer (pH 6.0) and microwaved for antigen removal for 10 min. Endogenous peroxidase activity was eliminated after immersing the slides in H_2_O_2_ (3%). After blocking nonspecific binding by incubating in 5% bovine serum albumin for 30 min, the sections were incubated overnight at 4℃ with rabbit anti-CK5/CK6 (1:200 dilutions; MA5-12429, Invitrogen, CA, USA), anti-CK7 (1:400; M7018, Dako, CA, USA), and anti-TTF1 (1:200; ab76013, MA, USA), followed by incubation with a biotinylated secondary antibody (diluted at 1:500) at 37 °C for 60 min. The slides were stained with diaminobenzidine and counterstained with hematoxylin. Finally, all slides were dehydrated by incubating with alcohol and further immersed in xylene for 5 min. All images were taken using an inverted fluorescence microscope (Olympus, Japan).

### siRNA transfection

The LUAD cells (A549 and PC9) and LUAD organoids were seeded on six-well plates at a density of 3 × 10^5^ cells/well. Twenty-four hours after cell seeding, the cells were transfected with siRNAs using Lipofectamine 2000 reagent (Invitrogen, CA, USA) following the manufacturer’s protocols. The sequences of siRNAs targeting CDK1, CCNB2, and CDC25A are listed in Additional file 1: Table S1.

### Cell counting kit 8 assay

After transfection for 48 h, the cancer cells were plated in 96-well plates (5 × 10^3^ cells/well). The cell proliferation ability was examined using a cell counting kit 8 (CCK-8) assay (Dojindo, Beijing, China) after 1–7 days following the manufacturer’s protocol. Finally, the absorbance was measured at 450 nm, and every experiment was performed in triplicate.

### Colony formation assay

A total of 1000 cells were seeded onto 6-well plates. After culturing for 2 weeks in RPMI 1640 with 1% FBS, the colonies were washed using PBS and stained with crystal violet for 30 min at room temperature. Finally, the colonies were visualized and counted.

### Statistical analysis

Venn diagrams were plotted to show the intersected gene sets and genes. Kaplan–Meier survival analysis was conducted using the “survival” package (Version 2.41-3) in R. Forest plots also used to display the results. Using GraphPad Prism software (GraphPad Software Inc., CA, USA), the results from different experiments were compared using the Student *t* test and shown as the mean ± standard deviation. A significant difference was set at the *P* value < 0.05 [11].

## Results

### Comprehensive transcriptomic analysis of tumor tissues, tumor organoids, and normal tissues revealed the molecular mechanisms of LUAD and LUSC

**Fig. 1:**
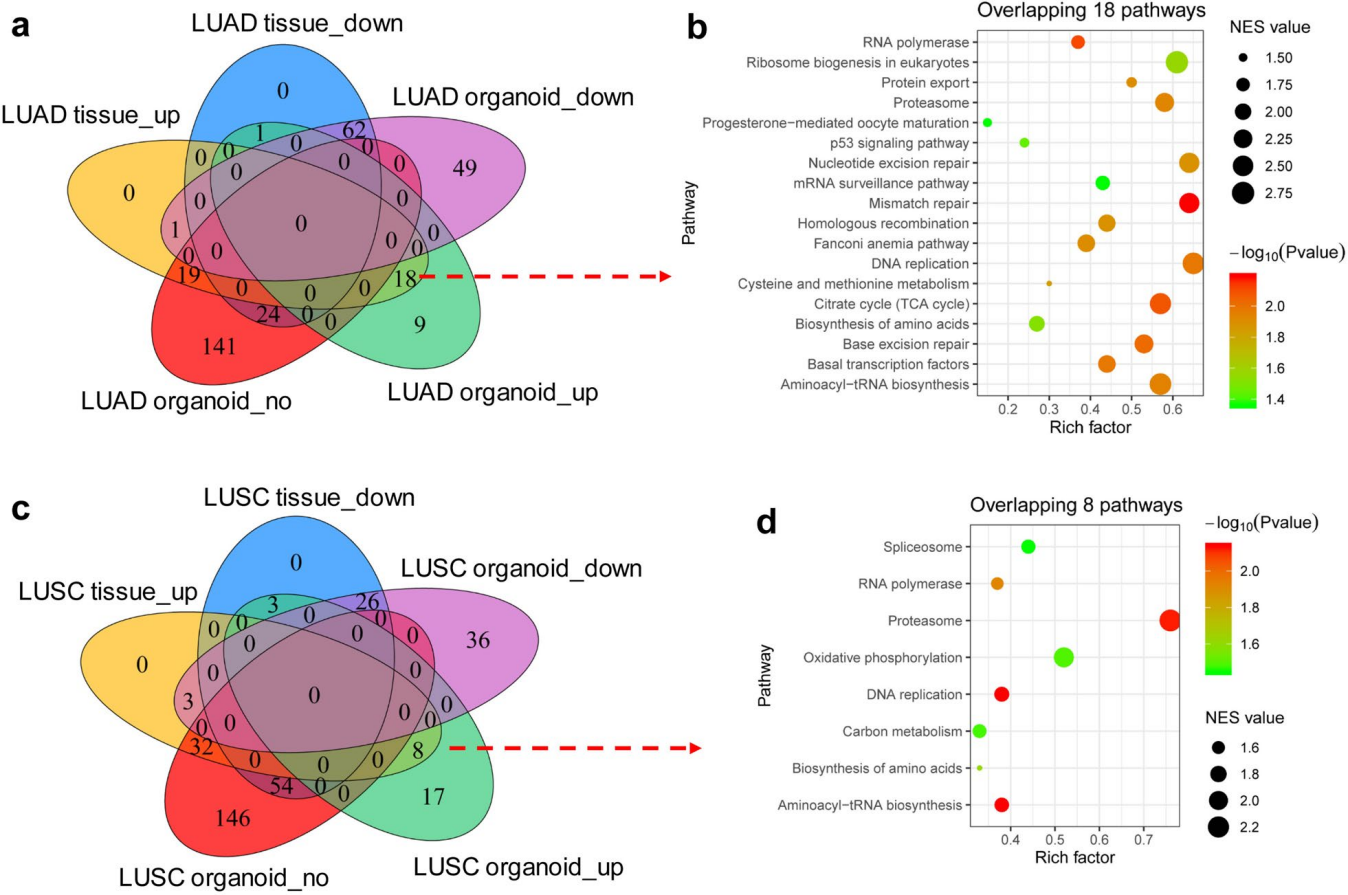
Transcriptomic analysis based on organoids and tissues revealed the mechanisms of LUAD and LUSC respectively. **a** Venn diagram showed the intersection of pathways enriched in LUAD tissues compared with normal tissues (58 paired LUAD tissues and normal tissues) and pathways enriched in LUAD organoids compared with LUAD tissues (6 paired LUAD organoids and tissues). **b** Bubble diagram showed 18 pathways enriched in LUAD tissues compared with normal tissues, which were also enriched in LUAD organoids compared with LUAD tissues. **c** Venn diagram showed the intersection of pathways enriched in LUSC tissues compared with normal tissues (49 paired LUSC tissues and normal tissues) and pathways enriched in LUSC organoids compared with LUSC tissues (2 paired LUSC organoids and tissues). **d** Bubble diagram showed eight pathways enriched in LUSC tissues compared with normal tissues, which were also enriched in LUSC organoids compared with LUSC tissues. *up* upregulated genes, *down* downregulated genes; *no* no difference

GSEA analysis was performed between paired LUAD and normal tissues (*n* = 58), and paired LUAD organoid and LUAD tissues (*n* = 6). After the intersection, 18 pathways enriched in LUAD tissues compared with normal tissues were obtained and simultaneously enriched in LUAD organoids compared with LUAD tissues (Fig. 1a). The organoid culture could purify tumor cells from tumor tissues. Therefore, the aforementioned 18 signaling pathways might be crucial mechanisms involved in the carcinogenesis and development of LUAD, which were mainly related to the P53 signaling pathway, base mismatch repair, and DNA replication (Fig. 1b). In the same way (Fig. 1c, d), eight pathways enriched in LUSC tissues compared with normal tissues were obtained and simultaneously enriched in LUSC organoids compared with LUSC tissues (Fig. 1c). For example, these pathways were mainly related to the gene ontology (GO) terms of DNA replication and spliceosome (Fig. 1d).

**Fig. 2:**
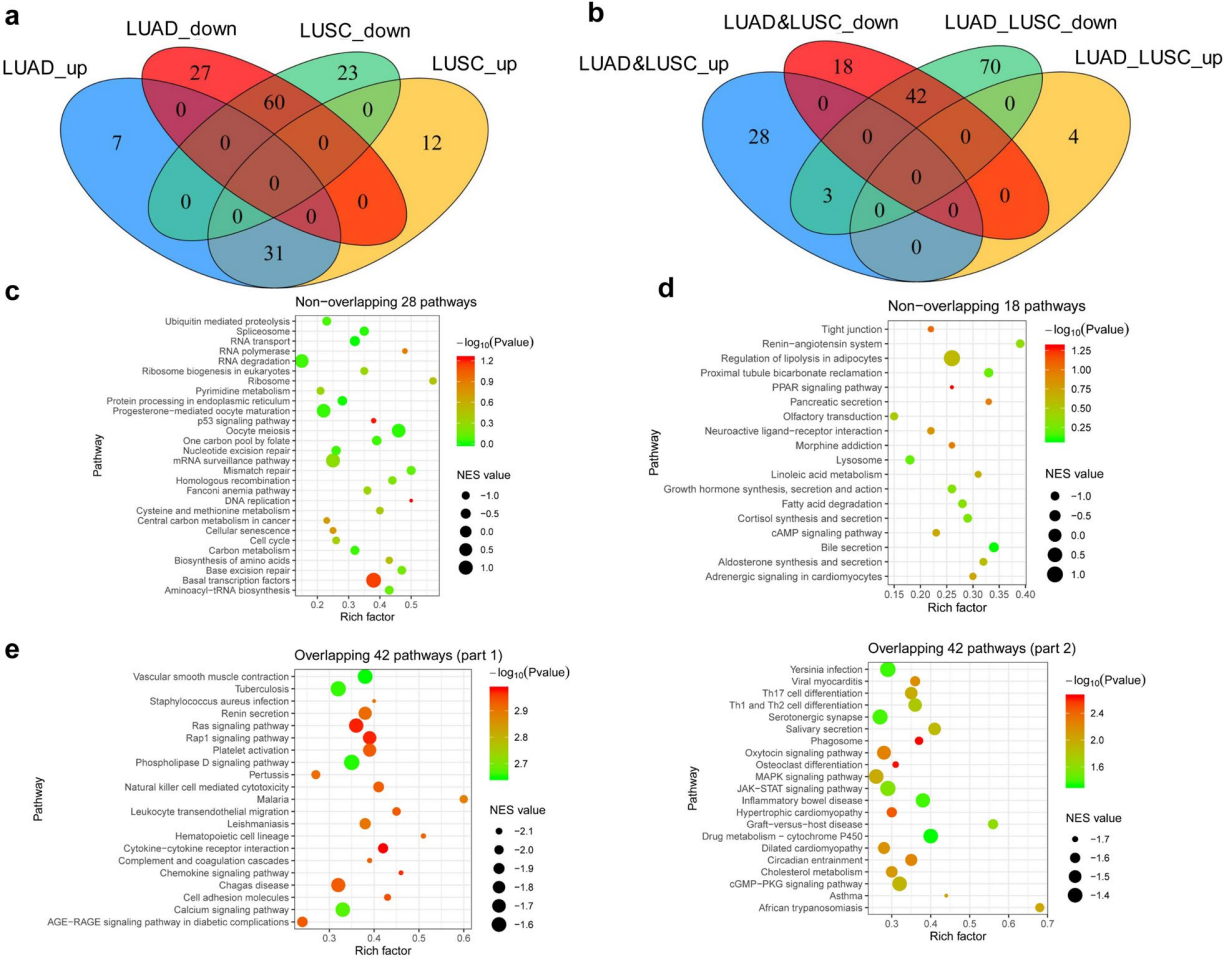
Transcriptomic analysis based on organoids and tissues identified different mechanisms in LUAD and LUSC. **a** Venn diagram showed the intersection of pathways enriched in LUAD (n = 58) and LUSC (n = 49) tissues compared with the matched normal tissues. **b** Venn diagram showed the intersection of pathways enrichment between LUAD (n = 6) and LUSC (n = 2) organoids, which were also enriched in the lung cancer tissues compared with normal tissues. **c-e** Venn diagram showed the overlapping pathways upregulated in NSCLC tissues but with no difference between LUAD (n = 6) and LUSC (n = 2) organoids (**c**), 18 signaling pathways downregulated in NSCLC tissues but with no difference between LUAD (n = 6) and LUSC (n = 2) organoids (**d**), and 42 signaling pathways downregulated in NSCLC tissues, which were further downregulated in LUAD organoids compared with LUSC organoids (**e**). up: upregulated genes; down: downregulated genes

A Venn diagram was drawn to obtain the positively and negatively correlated gene sets in LUAD and LUSC tissues compared with normal tissues so as to further explore the similar and different mechanisms involved in the carcinogenesis and development of LUAD and LUSC. A total of 31 upregulated pathways and 60 downregulated pathways were obtained (Fig. 2a). Meanwhile, the pathway enrichment between LUAD and LUSC organoids was also assessed using GSEA analysis. By intersecting with the aforementioned upregulated 31 pathways and downregulated 60 pathways (Fig. 2b), the following pathways were obtained: 28 signaling pathways, which were upregulated in LUAD and LUSC tissues but with no difference between LUAD and LUSC organoids; 18 signaling pathways, which were downregulated in NSCLC tissues but with no difference between LUAD and LUSC organoids; and 42 signaling pathways downregulated in LUAD and LUSC tissues and further downregulated in LUAD organoids compared with LUSC organoids (Fig. 2c–e). The analysis results showed that LUAD and LUSC had similar carcinogeneses and development mechanisms. For example, the upregulated pathways in LUAD and LUSC were P53 signaling pathway, base mismatch repair, and DNA replication (Fig. 2c), and the downregulated pathways in LUAD and LUSC were cAMP signaling pathway and PPAR pathway (Fig. 2d). They also revealed the downregulated pathways after comparing LUAD with LUSC organoids, although these pathways were downregulated in LUAD and LUSC tissues by comparing with normal tissues, such as immune-related pathways, inflammation-related pathways, MAPK signaling pathways, and Rap1 signaling pathways (Fig. 2e).

### Identification of genes involved in LUAD and LUSC carcinogenesis and development

**Fig. 3:**
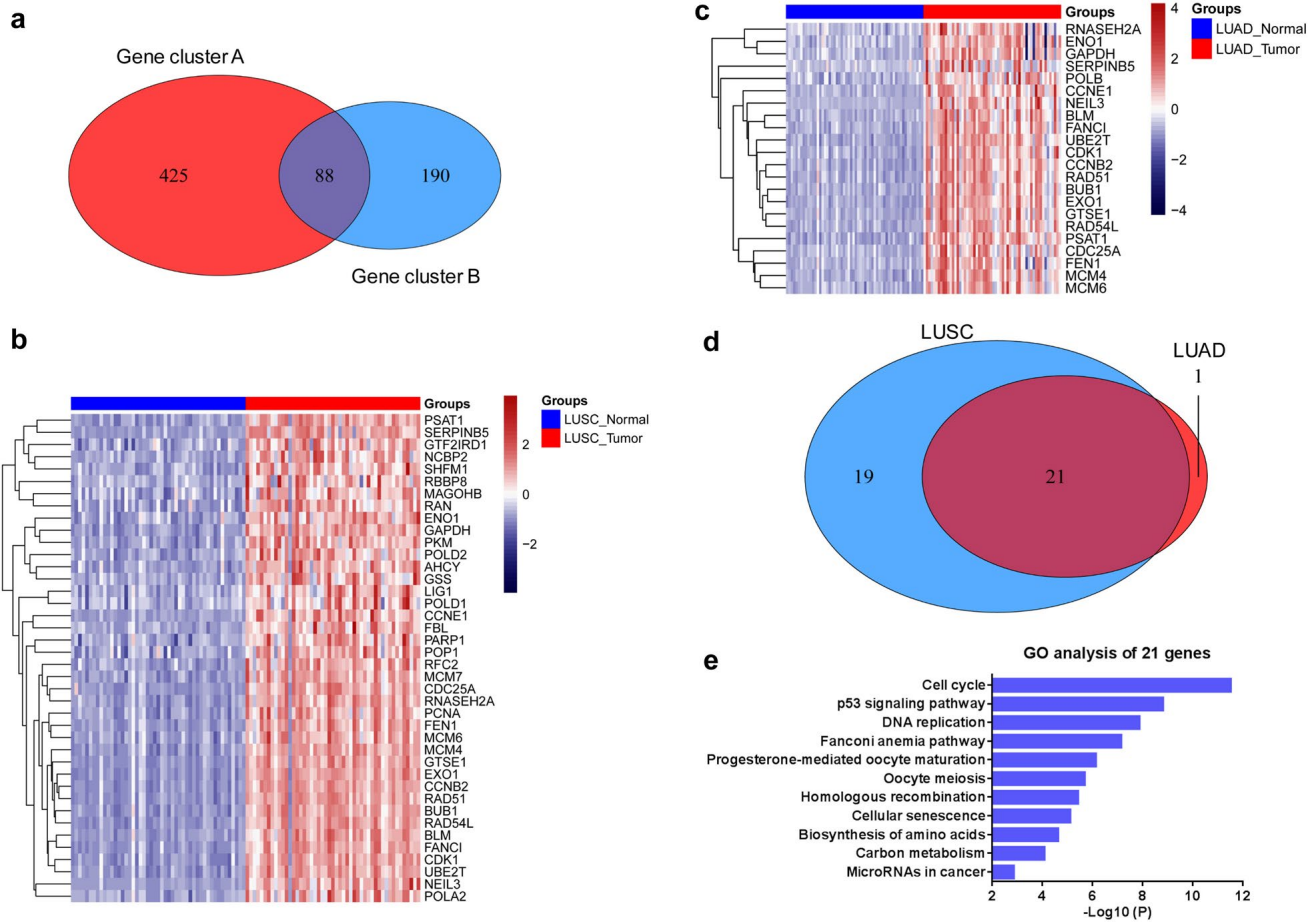
Screening of lung adenocarcinoma-related genes based on organoid and tissue analysis. **a** Intersection of the genes enriched in the pathways in Fig. 1B and Fig. 2C was taken, and 88 genes were obtained. Gene cluster A: genes upregulated in LUAD tissue and organoid; Gene cluster B: genes upregulated in LUAD and LUSC tissue but no difference between LUAD and LUSC organoid. **b**, **c** Heatmaps showed 40 genes upregulated in LUSC (n = 49) tissues compared with the matched normal tissues (**b**, LogFC > 1, *P* < 0.001) and 22 genes upregulated in LUAD (n = 58) tissues compared with the matched normal tissues (**c**, LogFC > 1, *P* < 0.001). **d** Venn diagram showed the overlapping 21 genes upregulated in LUAD (n = 58) and LUSC (n = 49) tissues compared with the matched normal tissues. **e** Pathways analysis by Metascape revealed the enriched pathways of the overlapping 21 genes

The aforementioned results indicated that LUAD and LUSC shared similar mechanisms of carcinogenesis and development. The intersection of the genes in the 18 signaling pathways in Fig. 1b and 28 signaling pathways in Fig. 2c was taken to further reveal the hub genes taking part in the malignant biological behaviors of LUAD and LUSC; 88 overlapping genes were obtained (Fig. 3a). The *t* test results showed that 40 of 88 genes were significantly upregulated (*P* < 0.001, LogFC > 1, Fig. 3b) and 22 of 88 genes were significantly upregulated in LUAD tissues compared with normal tissues (*P* < 0.001, LogFC > 1, Fig. 3c). We also plotted heatmaps to displayed these genes expression between all 525 LUADs and 58 normal tissues (Additional file 2: Figure S1a), or between all 501 LUSCs and 49 normal tissues (Additional file 2: Figure S1b) in TCGA database. A Venn diagram was plotted to obtain 21 overlapping genes, which were simultaneously upregulated in LUAD and LUSC tissues (Fig. 3d). Pathways analysis by Metascape revealed that these 21 genes were mainly related to the P53 signaling pathway, DNA replication, and cell cycle (Fig. 3e).

**Fig. 4:**
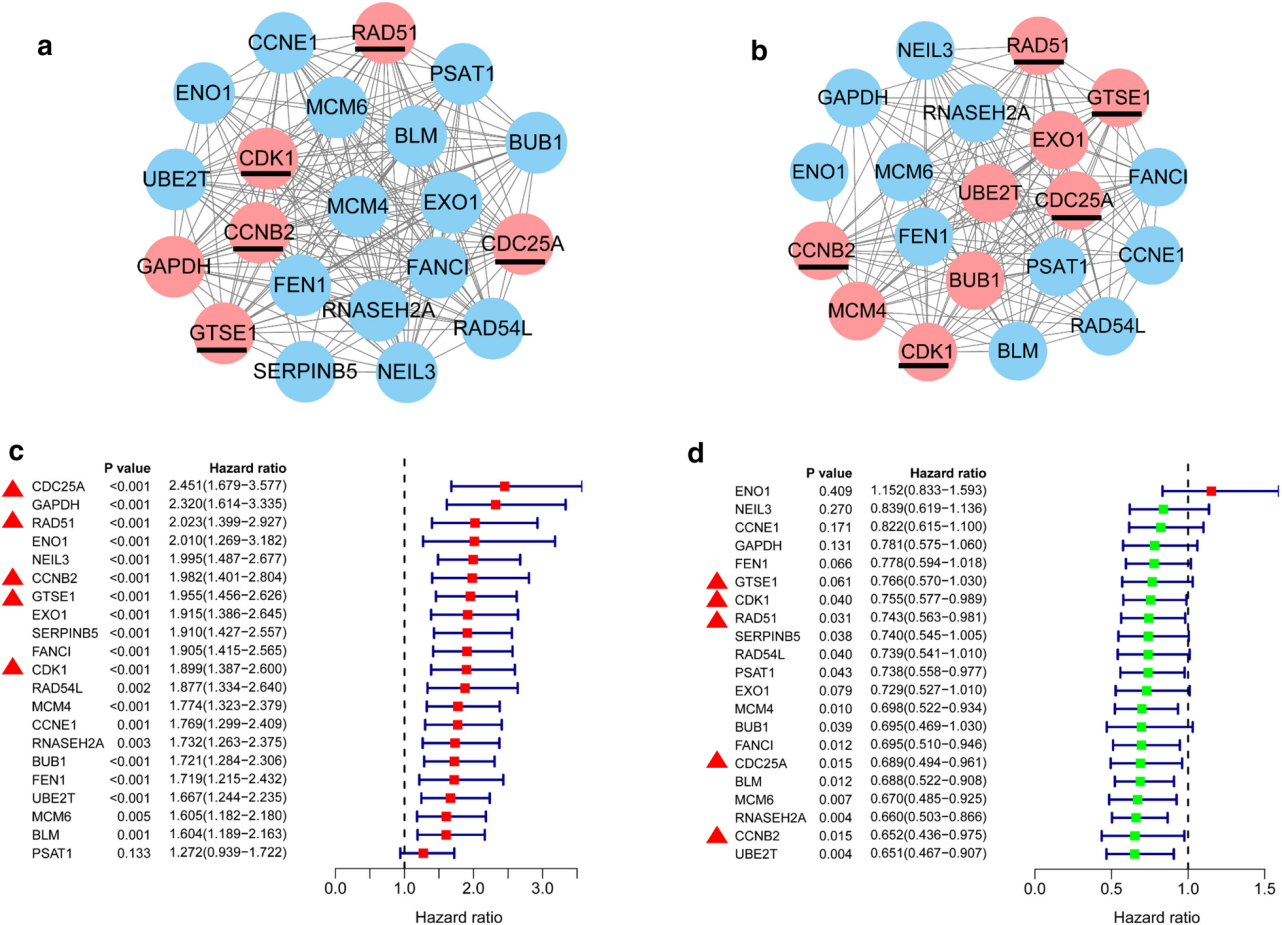
Identification of hub genes involved in LUAD and LUSC carcinogenesis and development. **a**, **b** Using the transcriptome data of LUAD (**a** n = 525) and LUSC (**b** n = 501) in the TCGA database, gene co-expression network analysis was performed to identify 5 overlapping hub genes from the aforementioned 21 overlapping genes: RAD51, CDK1, CCNB2, CDC25A, and GTSE1. **c**, **d** Univariate prognostic analysis of the aforementioned 21 genes in LUAD (**c**) and LUSC (**d**)

**Fig. 5:**
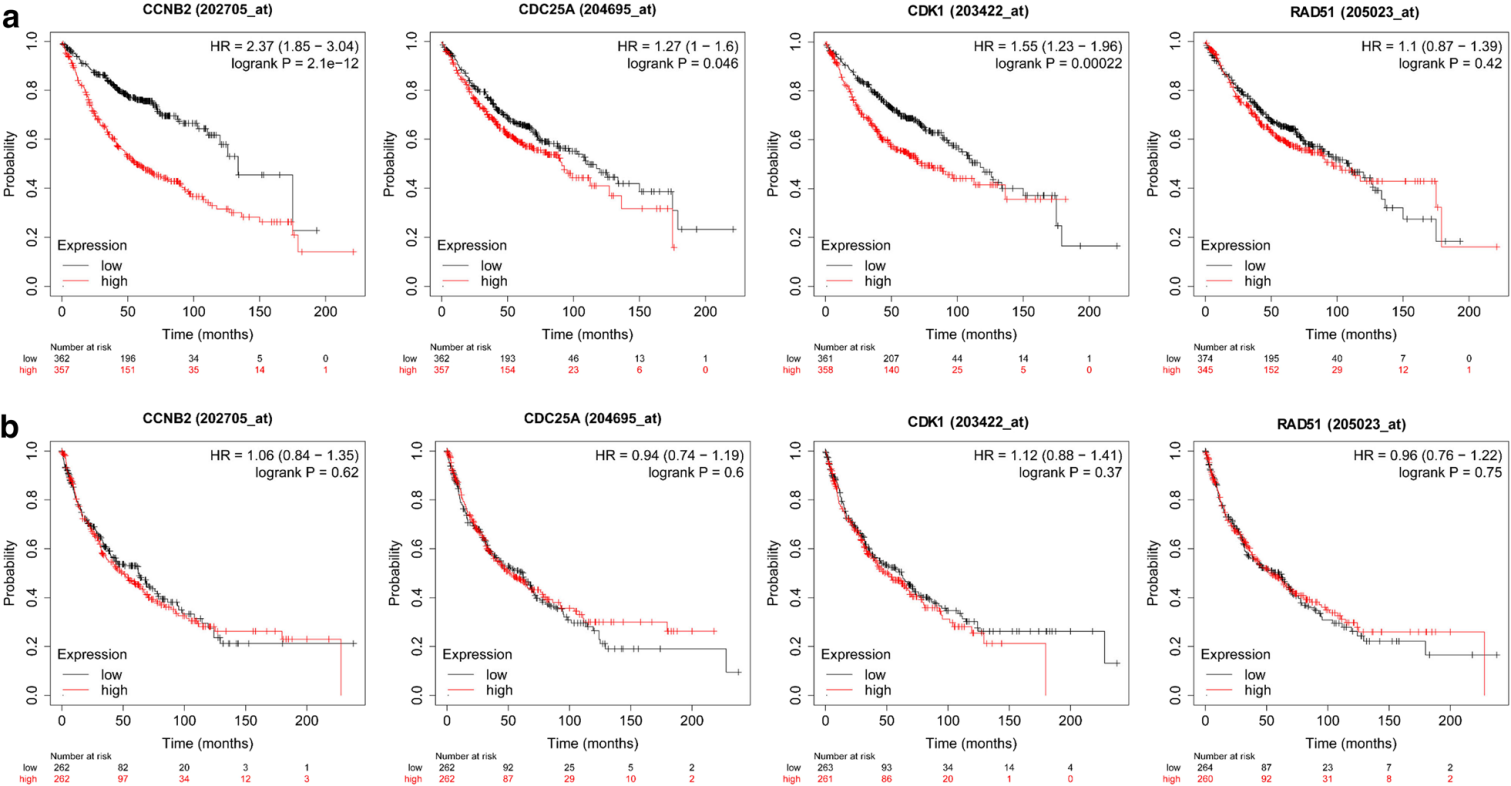
Validation of prognostic discrimination of RAD51, CDK1, CCNB2, and CDC25A in other datasets. **a**, **b** Prognostic analysis using the Kaplan–Meier Plotter database showed that the expression of CDK1, CCNB2, and CDC25A positively correlated with the poor prognosis of LUAD (**a** n = 719), but not significantly correlated with the prognosis in LUSC (**b** n = 524)

The transcriptomic data of LUAD (Fig. 4a) and LUSC (Fig. 4b) tissues in the TCGA database were used to perform gene co-expression network analysis so as to further screen for key genes and five overlapping key genes RAD51 recombinase (RAD51), CDK1, CCNB2, CDC25A, and G2 and S-phase expressed 1 (GTSE1) were identified. The prognostic analysis found that the expression of the aforementioned 21 genes in LUAD positively correlated with poor prognosis (Fig. 4c), but in LUSC, these genes were associated with good prognosis (Fig. 4d). These results indicated that the mechanisms involved in the carcinogenesis and development of LUAD were still quite different from those of LUSC, suggesting that the following further research about LUAD and LUSC should be treated separately. The Kaplan–Meier Plotter database was used for further verification, revealing that the expression of CDK1, CCNB2, and CDC25A positively correlated with the poor prognosis of LUAD (Fig. 5a), but no significance in LUSC (Fig. 5b).

### Effects of the knockdown of CDK1, CCNB2, and CDC25A on the proliferative ability of LUAD cell lines

**Fig. 6:**
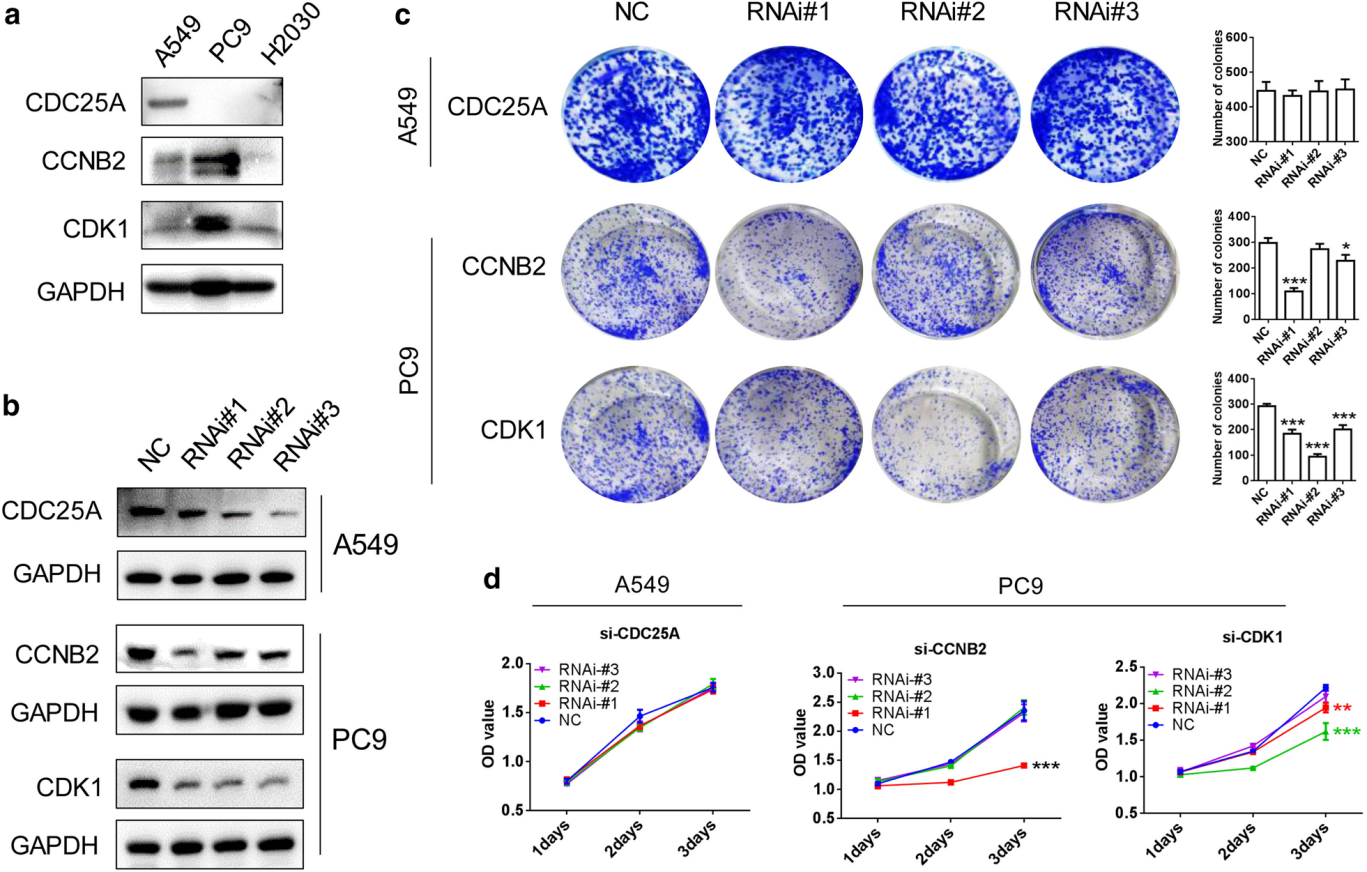
Effects of CDK1, CCNB2, and CDC25A expression on proliferation of LUAD cell lines in vitro. **a** Expression of CDK1, CCNB2, and CDC25A in LUAD cell lines A549, PC9, and H2030. **b** Using siRNA, CDK1, CCNB2, and CDC25A were knocked down in LUAD cell lines. **c**, **d** Colony formation assays (**c**) and CCK-8 assays (**d**) were used to detect the effects of CDK1, CCNB2, and CDC25A knockdown on the proliferation of LUAD cell lines

Western blot was used to detect the expression of CDK1, CCNB2, and CDC25A in LUAD cell lines A549, PC9, and H2030 (Fig. 6a). The cell line A549 with the high expression of CDC25A and the PC9 cell line with the high expression of CDK1 and CCNB2 were selected for the subsequent experiments. After knocking down CDK1, CCNB2, and CDC25A by siRNA transfection (Fig. 6b), the colony formation assays and CCK-8 assays were performed. It was found that CDK1 and CCNB2 knockdown inhibited the cloning ability and proliferation of LUAD cell line PC9, but CDC25A knockdown had no effect on the clone formation and proliferation ability of LUAD cell line A549 (Fig. 6c, d).

### Knockdown of CDK1, CCNB2, and CDC25A inhibited LUAD organoid growth

**Fig. 7:**
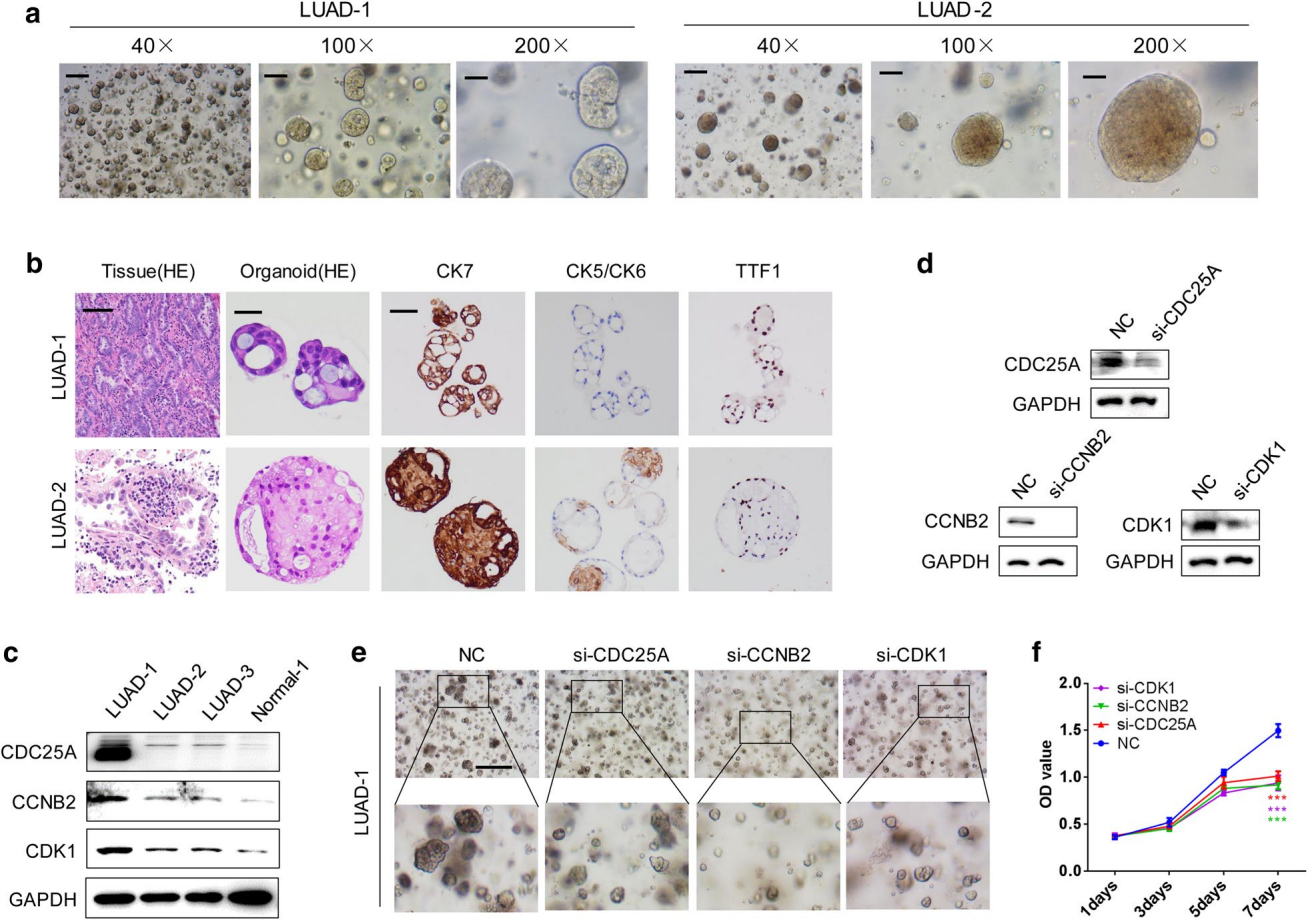
Effects of CDK1, CCNB2, and CDC25A expression on the growth of LUAD organoids. **a** Two LUAD-derived organoids LUAD-1 and LUAD-2 were successfully constructed. **b** H&E staining of LUAD tissues and derived organoids. IHC analysis of CK5/CK6, CK7, and TTF1 expression in LUAD-1 and LUAD-2 organoids. **c** Expression of CDK1, CCNB2, and CDC25A in lung organoid and LUAD organoids by Western blot analysis. **d** By siRNA transfection, the expression of CDK1, CCNB2, and CDC25A was knocked down in LUAD-1 organoids. **e**, **f** The effects of CDK1, CCNB2, and CDC25A knockdown on the growth of LUAD organoids observed under a microscope (**e**) and using CCK-8 assays (**f**)

Two LUAD organoids LUAD-1 and LUAD-2 were successfully extracted and cultured using fresh postoperative tissues (Fig. 7a and Additional file 3: Figure S2). LUAD organoid-derived tissues and organoids were stained with H&E, and CK5/CK6, CK7, and TTF1 IHC staining of organoids was used to identify the source of organoids (Fig. 7b and Additional file 3: Figure S2). Organoids derived from LUAD had high levels of CK7 and TTF1 but a low level of CK5/CK6 (Fig. 7b). LUAD organoids showed the higher expression of CDK1, CCNB2, CDC25A than normal lung organoid Normal-1 by Western blot analysis, especially for LUAD-1 (Fig. 7c). Further, siRNA was used to successively knock down CDK1, CCNB2, and CDC25A of LUAD-1 organoids (Fig. 7d). CDK1, CCNB2, and CDC25A knockdown could slow down the growth of LUAD organoids (Fig. 7e), and the proliferation ability was also weakened, as detected using CCK-8 assays (Fig. 7f). Similarly, knockdown of CDK1 and CCNB2 also slowed down LUAD-2 growth (Additional file 4: Figure S3a–c).

## Discussion

Lung cancer is the leading cause of cancer-related mortality worldwide. However, 40%–60% of patients with lung cancer are in advanced stages when they are diagnosed [16]. Therefore, novel biomarkers need to be urgently developed to predict prognosis and targets for lung cancer treatment. Patient-derived organoids could be used to model various human pathologies “in a dish,” and well study the mechanisms involved in tumor carcinogenesis and development [17]. In this study, a systemic analysis was performed to reveal CDK1, CCNB2, and CDC25A as the hub genes; the expression of these genes increased in tumor tissues and predicted poor prognosis in LUAD. Finally, the experiments using cancer cell lines and organoids confirmed that CDK1, CCNB, and CDC25A could induce LUAD proliferation and colony formation. Therefore, the potential roles of CDK1, CCNB2, and CDC25A in diagnosing and treating LUAD were revealed.

GSEA analysis was performed between normal tissues and tumor tissues, or normal and cancer organoids of LUAD and LUSC. The GO terms of the P53 signaling pathway, cell cycle, and DNA replication were upregulated in both LUAD and LUSC, which played crucial roles in lung cancer carcinogenesis and progression [18–20]. Further screening by establishing gene co-expression networks and preforming prognostic analysis identified CDK1, CCNB2, and CDC25A as the hub genes involved in lung cancer carcinogenesis and development. CDK1 is a cyclin kinase that can lead to malignant cell proliferation after activation [21]. Fu et al. reported that CDK1 could be used as a biomarker to predict the postoperative brain metastasis of LUAD [22]. Wang et al. found that CDK1 could promote cell viability, colony-forming ability, migration, and invasion of lung cancer [23]. CDK1 knockdown suppressed the proliferation of LUAD cells and organoids, revealing its crucial role in LUAD. In LUAD, CCNB2 could promote proliferation, migration, invasion, and cell cycle G0/G1 phase transition of cancer cells [19]. CDC25A, a member of the CDC25 family of phosphatases, is required for progression from the G1 phase to the S phase of the cell cycle. Some studies indicated CDC25A as an oncogene; however, its exact role in oncogenesis in LUAD has not been fully demonstrated [24–26]. This study found that CDC25A knockdown did not obviously suppress the proliferation and colony formation of LUAD cell line A549, but inhibited the growth of LUAD organoids. CCNB2, a member of cyclin family proteins, plays a critical role in the progression of G2/M transition [27]. Niemira et al. identified CCNB2 as a key gene involved in lung cancer progression by weighted gene co-expression network analysis using large-scale transcriptional profiling [28]. Wang et al. reported that the knockdown of CCNB2 attenuated the proliferation, migration, invasion, and cell cycle of LUAD cells [19]. Similarly, the downregulation of CCNB2 not only apparently attenuated LUAD cell proliferation and colony formation but also suppressed LUAD organoids growth.

An increasing number of studies indicated that the use of an organoid-based model could help reveal novel cancer driver genes [10, 29, 30]. Among the aforementioned three hub genes, CDK1 and CCNB2 promoted LUAD growth in this study, which were previously implicated in LUAD progression using cancer cell line models. In vitro experiments using cell lines and organoids indicated that CDC25A knockdown could not inhibit LUAD cell proliferation and colony formation but suppressed LUAD organoid growth. Using a recellularized human colon model, Chen et al. revealed some genes involved in invasion in colon cancer, such as ASXL2, CAMTA1, DDX20, FXR1, MITF, and PAX7 [10]. The results of this study also indicated organoid as a good model used to screen hub genes involved in LUAD progression and examine LUAD biology.

In conclusion, the bioinformatics analysis was performed in this study to screen and identify the hub genes involved in LUAD progression. Further in vitro experiments using cell lines and organoid models confirmed that CDK1, CCNB2, and CDC25A could induce LUAD growth. Therefore, LUAD organoid served as a good model to examine LUAD biology.

## Supplementary Information

**Additional file 1: Table S1.** Sequences of siRNAs used in this study.

**Additional file 2: Figure S1.** Heatmaps to display the expression of 40 upregulated genes between all 525 LUADs and 58 normal tissues (**a**), or between all 501 LUSCs and 49 normal tissues (**b**) in TCGA database.

**Additional file 3: Figure S2.** H&E staining of LUAD organoid (LUAD-3) and normal organoid (Normal-1).

**Additional file 4: Figure S3.** Effects of CDK1, CCNB2, and CDC25A expression on the growth of LUAD-2 organoids. **a** CDK1, CCNB2, and CDC25A were knocked down in LUAD-2 organoids by siRNA transfection. The effects of CDK1, CCNB2, and CDC25A knockdown on the growth of LUAD-2 organoids were assessed under a microscope (**b,** 20 ×) and using CCK-8 assays (**c**).

## Data Availability

The data used to support the findings of this study are included within the article.

## Abbreviations

GSEA: Gene set enrichment analysis
LUAD: Lung adenocarcinoma
LUSC: Lung squamous cell carcinoma
TCGA: The Cancer Genome Atlas
GEO: Gene Expression Omnibus
CDK1: Cyclin-dependent kinase 1
CCNB2: Cyclin B2
CDC25A: Cell division cycle 25A
RAD51: RAD51 recombinase
GTSE1: S-phase expressed 1
OS: Overall survival
H&E: Hematoxylin–eosin
IHC: Immunohistochemistry
