# Supplementary material for: Transcriptomic analysis of tumor tissues and organoids reveals the crucial genes regulating the proliferation of lung adenocarcinoma
Source: J Transl Med. 2021 Aug 26;19:368. doi: 10.1186/s12967-021-03043-6 (PMC8393455; doi:10.1186/s12967-021-03043-6)
Supplement: Supplementary file 1 — Additional file 1: Table S1. Sequences of siRNAs used in this study. [file 12967_2021_3043_MOESM1_ESM.docx]

Table S1. Sequences of siRNAs used in this study.

| Gene name | Sense | Antisense |
| --- | --- | --- |
| CDK1 |  |  |
| RNAi#1 | GGGGUUCCUAGUACUGCAATT | UUGCAGUACUAGGAACCCCTT |
| RNAi#2 | GGCACUGAAUCAUCCAUAUTT | AUAUGGAUGAUUCAGUGCCTT |
| RNAi#3 | CAGGACUAUAAGAAUACAUTT | AUGUAUUCUUAUAGUCCUGTT |
| CCNB2 |  |  |
| RNAi#1 | GGCCAAGAAUGUGGUGAAATT | UUUCACCACAUUCUUGGCCTT |
| RNAi#2 | CCAGUGAUUUGGAGAAUAUTT | AUAUUCUCCAAAUCACUGGTT |
| RNAi#3 | GGUGCAUUAUCAUCCUUCUTT | AGAAGGAUGAUAAUGCACCTT |
| CDC25A |  |  |
| RNAi#1 | GGCCAUUGGACAGUAAAGATT | UCUUUACUGUCCAAUGGCCTT |
| RNAi#2 | GCCCAUUGUACCUACUGAUTT | AUCAGUAGGUACAAUGGGCTT |
| RNAi#3 | GCCAGUAAGACCUGUAUCUTT | AGAUACAGGUCUUACUGGCTT |
